# Correlation between delivered radiation doses to the brainstem or vestibular organ and nausea & vomiting toxicity in patients with head and neck cancers – an observational clinical trial

**DOI:** 10.1186/s13014-017-0846-4

**Published:** 2017-07-04

**Authors:** Kilian Schiller, Hanno Martin Specht, Bernhard Haller, Daniela Hallqvist, Michal Devecka, Aaron Becker von Rose, Stephanie Elisabeth Combs, Steffi Pigorsch

**Affiliations:** 1 Department of Radiation Oncology, Klinikum rechts der Isar der Technischen Universität München, Ismaninger Str. 22, 81675 Munich, Germany; 2 Institute of Medical Statistics and Epidemiology, Klinikum rechts der Isar der Technischen Universität München, Ismaninger Str. 22, 81675 Munich, Germany

**Keywords:** Head and neck cancer, IMRT, VMAT, Toxicity, Brainstem

## Abstract

**Objective:**

Today intensity modulated radiation therapy (IMRT) can be considered the standard of care in patients with head and neck tumors. IMRT treatment plans are proven to reduce acute treatment related side effects by optimal sparing of organs at risk (OAR). At the same time, areas that were out of the former 3D fields now receive low radiation doses. Amongst those areas the brainstem (BS) and the vestibular system (VS) are known to be physiologically connected to nausea and vomiting (NV). In our study we tried to find out, if doses to these areas are linked to NV.

**Material & Methods:**

NV were assessed at different time points during treatment in 26 patients leading to 98 documented toxicity scores that were later correlated to dose deposition in the described areas. Patients were either treated with normo-fractionated or simultaneously integrated boost IMRT plans in a curative approach. Subareas of the BS as well as the VS were delineated. Toxicity was rated based on the common toxicity criteria (CTCAE Version 4.0). Other factors such as age, gender, chemotherapy, location of the tumor, irradiated volume and unilateral dose to the VS were taken into account and analyzed also.

**Results:**

The majority (65.4%) of our patients experienced an episode of NV at least once during treatment. NV was more frequent when treating the oropharyngeal region compared to the hypopharyngeal region, as well as when patients were female and/ or of a younger age. Nevertheless, upon statistical analysis (ROC analysis, ‘within/ between analysis’) no significant association between delivered doses to subareas and toxicity could be demonstrated.

**Conclusion:**

In our analysis, no significant correlation between radiation dose to the BS or the VS and the occurrence of NV could be found. Therefore, until conclusive data are available, we recommend to rely on the published data regarding OAR tolerance within the BS and not to compromise on dose coverage.

**Electronic supplementary material:**

The online version of this article (doi:10.1186/s13014-017-0846-4) contains supplementary material, which is available to authorized users.

## Background

Today Intensity modulated radiation therapy (IMRT) can be considered the standard of care in patients with head and neck (HN) tumors [1]. Patients with HN cancers who were treated with IMRT experienced significant improvements in cause specific survival compared with patients treated with non-IMRT techniques [2]. This suggests there may be benefits by IMRT in cancer outcomes, in addition to toxicity reduction, for this patient population. IMRT treatment plans reach a higher PTV-conformity compared to 3D plans and possibly allow dose escalation by reducing radiation doses to surrounding organs at risk [3, 4].

Today IMRT can be delivered using different technical concepts, e.g. step and shoot IMRT, volumetric modulated arc treatment (VMAT) or helical tomotherapy. In IMRT concepts, a range of treatment angels can be present and based on inverse planning algorithms, the optimal dose distribution is calculated depending on the prescribed dose coverage in the planning target volume (PTV) and dose constraint in the organs at risk (OAR). Hence the dose bath and the volume of normal tissue receiving low dose radiation can be increased, which may lead to previously unknown and unexpected side effects [5, 6].

In HN cancer treatment, this “low dose spill” into non target structures such as the brainstem (BS) or the vestibular system (VS) is possibly linked to an increase in nausea and vomiting toxicities (NV), as the Area postrema (AP) in the brain stem is a known trigger zone for vomiting [5, 7].

Out of all HN patients treated with radiation 40% suffer from emesis with conventional radiation techniques, whereas some authors suggest that numbers tend to be higher in patients treated with IMRT [8, 9].

Furthermore, doses to the peripheral labyrinthine and vestibules have previously been linked with vertigo and could consequently also lead to NV toxicity. Although it has been described as a late complication as a sequel of radiation otitis media, the possibility of it as an acute reaction seems only logic [10]. Therefore, it has been described that the vestibules should be labeled as sensitive OARs [11].

Nonetheless, it remains unclear, whether NV is directly linked to the radiation dose applied to the previously mentioned areas. In our study we tried to find out by dosimetric evaluation, if doses to subareas of the BS or the VS are linked to NV and if so; is there a threshold for its appearance?

## Materials and Methods

Twenty six patients with HN tumors were treated between 2012 and 2014 and retrospectively evaluated in this study (patient characteristics are summarized in Table 1). All patients were treated with standard of care normo-fractionated (84.6%) or SIB-IMRT (15.4%) plans in a curative approach. After the consent of an interdisciplinary tumor-board, patients were treated with concurrent IMRT-chemotherapy or with IMRT alone. Cumulative doses ranged between 60 and 70.4 Gray (Gy) (median dose 65 Gy) at single doses ranging between 1.7 and 2.2 Gy for SIB concepts with otherwise normo-fractionated schemes (2 Gy up to 50 Gy, then 2 Gy to a cumulative dose to the boost volume of 70 Gy). Four patients (15.4%) received a simultaneously integrated boost (SIB) to the primary tumor and to metastatic lymph nodes. IMRT or dynamic arc treatment planning was performed using Eclipse planning software (Varian Medical Systems, Palo Alto, CA) by experienced medical physicists.

**Table 1:** Patient characteristics

Gender	
Female	7 (27%)
Male	19 (73%)
Age (years)	
Median	62.5
Range	24–82
Primary sites (no. pts.)	
Oropharynx	15 (57.7%)
Hypopharynx	8 (30.8%)
Trans-regional	3 (11.5%)
(r) c/p T – Tumor size	
0	1 (3.8%)
1	1 (3.8%)
2	9 (34.6%)
3	9 (34.6%)
4	6 (23.2%)
(r) c/p N – Nodal Status	
0	8 (30.8%)
1	3 (11.5%)
2a	2 (7.7%)
2b	4 (15.4%)
2c	9 (34.6%)
M - Metastases	
0	26 (100%)
G – Grading	
1	2 (7.7%)
2	14 (53.8%)
3	10 (38.5%)
Concomittant chemotherapy (no. pts.)	13 (50%)
Cisplatin	12 (46.2%)
Paclitaxel	1 (3.8%)

For every patient a planning CT scan after manufacturing an individual thermoplastic mask fixation system in 3 mm slices after application of contrast media was performed. Nearly all patients got an additional MRI scan before treatment planning. MRI and CT data sets were merged for better target delineation and in the postoperative situation to exclude early relapse behind transplants and retropharyngeal lymph node involvement. For definitive normo-fractionated RT the GTV of the primary tumor (GTV-PT) and involved lymph nodes (GTV-LN) was delineated. The boost volume was generated by adding a margin of 10 mm around the GTV-PT and individual fit on each slice for anatomical boundaries considering set-up error was applied. To the GTV-LN 5–7 mm were added as margin to define the CTV. An additional margin of 3 mm was added for set-up error.

In the definitive SIB-IMRT the contouring was performed as explained: for the SIB high dose volume (2.2 Gy up to 70.4 Gy) GTV-PT/LN plus 5 mm margin, SIB intermediate dose volume (2.0 Gy up to 64.0 Gy) GTV-PT/LN plus 10 mm margin. The PTV for elective nodal irradiation was performed in all cases after the recommendations by Gregoire et al. The elective to treat lymph node drainage was either treated up to a total dose of 50 Gy definitive as well as adjuvant (single dose 2 Gy) or in a definitive SIB concept to 54,4 Gy (single dose 1.7 Gy). The involved lymph nodes received 70 Gy.

**Fig. 1:**
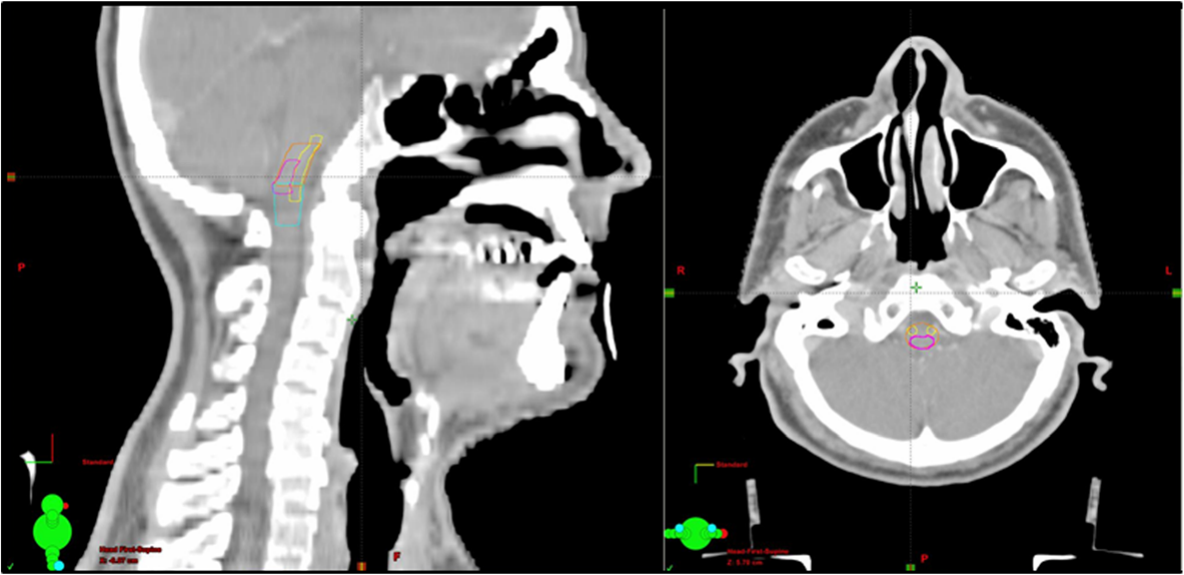
Contouring of the AP (*pink*), DVC (*yellow*) and the two boxes encompassing the brainstem (*mint* and *orange*)

In the adjuvant treatment situation the pre-operative GTV of primary tumor and involved lymph nodes were delineated after merging the pre-operative imaging to the planning CT, to define the pre-op GTV exactly information from pre-operative imaging, histology report and surgery report) were used. Two different adjuvant RT schemes were applied. Either normo-fractionation or a SIB-concept was used. The PTV for normo-fractionation was defined as described above. For the adjuvant SIB-concept for the SIB-volume (2.14 Gy up to 64.2 Gy) the pre-operative GTV-PT and GTV-LN got a margin of 10 mm. Flap transplants of the initial primary tumor region were included into the SIB-volume, it is described that the transplants tolerated RT well. The PTV definition was made in the same way as mentioned above. Conventional constraints to the OAR were applied (e.g. brainstem D_max_ 54Gy, Spinal cord D_max_ 45Gy). Additionally to the OAR, the AP, the dorsovagal complex (DVC) and the VS were contoured retrospectively using co-registered MRI imaging. The brainstem was divided into two halves (a cranial one (Box 1) and a caudal one (Box 2). No constraints were set on these additional structures, because they were not considered during treatment planning (Fig. 1).

Normo-fractionated radiation was performed with linear accelerators (Varian Medical Systems, Palo Alto, CA) using 6 MeV photons.

Concurrent chemotherapy (Cisplatin 20 mg/ m^2^ body surface/day 1–5 and day 29–33, Cisplatin 40 mg/m^2^ body surface/once weekly (up to six cycles) or (in one advanced case) Paclitaxel, 30 mg/m^2^ body surface/twice weekly, 4 cycles in total) was administered on our ward if indicated (in definitive chemo-irradiation and in the postoperative adjuvant/additive setting because auf extracapsular spread or R1-situation). Standard supportive care in the cisplatin protocol is the use of dexamethasone 16 mg IV and Granisetron 1 mg also IV in 100 ml NaCl 0.9% applied over 15 min prior to cisplatin. On the following 2 days patients get Dexamethason 16 mg per os and Metoclopramide three times a day 10 mg per os. Despite the antiemetics Pantoprazole 40 mg is given per os on each day.

At different time points during treatment (at the beginning, in the middle and last week of treatment) patients were evaluated concerning nausea and vomiting toxicity according to the National Cancer Institute’s Common Toxicity Criteria (v4.0) as standard procedure on ward rounds [12]. Symptoms recorded were intensity and duration of nausea, frequency of vomiting and accompanying symptoms such as pallor, cold sweating, tachycardia, hyper-salivation, gagging and neurological abnormalities. We also recorded if antiemetic drugs were given.

Later we correlated D_mean_ and D_max_ radiation doses of previously defined organs and volumes (AP, DVC, VS, Box 1 and 2) to present toxicity scores. Herein we evaluated 98 individual time points during radiotherapy.

For categorical data absolute and/or relative frequencies are presented. Within and between subjects correlation coefficients were determined in order to assess associations between administered doses and toxicity scores [13, 14]. A receiver operating characteristics (ROC) analysis was conducted to evaluate associations between doses administered to different sites and presence of any toxicity (toxicity score > 0). Due to multiple observations per patient, the method for correlated data proposed by Obuchowski was conducted [15]. Areas under the receiver operating characteristics curve (AUC) and corresponding 95% confidence intervals are presented. For all statistical tests, a significance level of 5% was used.

## Results

The majority (65.4%) of our patients experienced at least one episode of NV during treatment. In detail, 38.5% suffered mild NV (CTCAE I°), 15.4% moderate effects (CTCAE II°) and 11.5% severe ones (CTCAE III°). 12 out of 19 (63.2%) male patients had at least one episode of NV and 5 out of 7 (71.4%) of all female patients. Furthermore, younger patients were more likely to experience higher NV toxicity.

Concerning the tumor region, RT of oropharyngeal carcinomas were more prone to develop NV toxicity than other tumor regions. Patients with oropharyngeal cancer had at least one episode of NV in 73.3% of all cases, whereas for patients with hypopharyngeal cancer the part was 50%, respectively.

Twelve patients (46.2%) were treated in a definitive approach and the other 14 (53.8%) in an adjuvant/additive treatment-intention. Chemotherapy was administered if medically indicated and if overall clinical condition allowed it; exactly half of our patients received concurrent chemotherapy (CTx). We evaluated the association between chemo therapy and our outcome (the presence of at least one episode of NV). Eight of 13 patients (61.5%) under chemo therapy reported at least one episode with NV, while 9 of 13 patients (69.2%) without chemo therapy reported at least one episode of NV. This association was not statistically significant (chi squared test: *p* = 0.680).

**Fig. 2:**
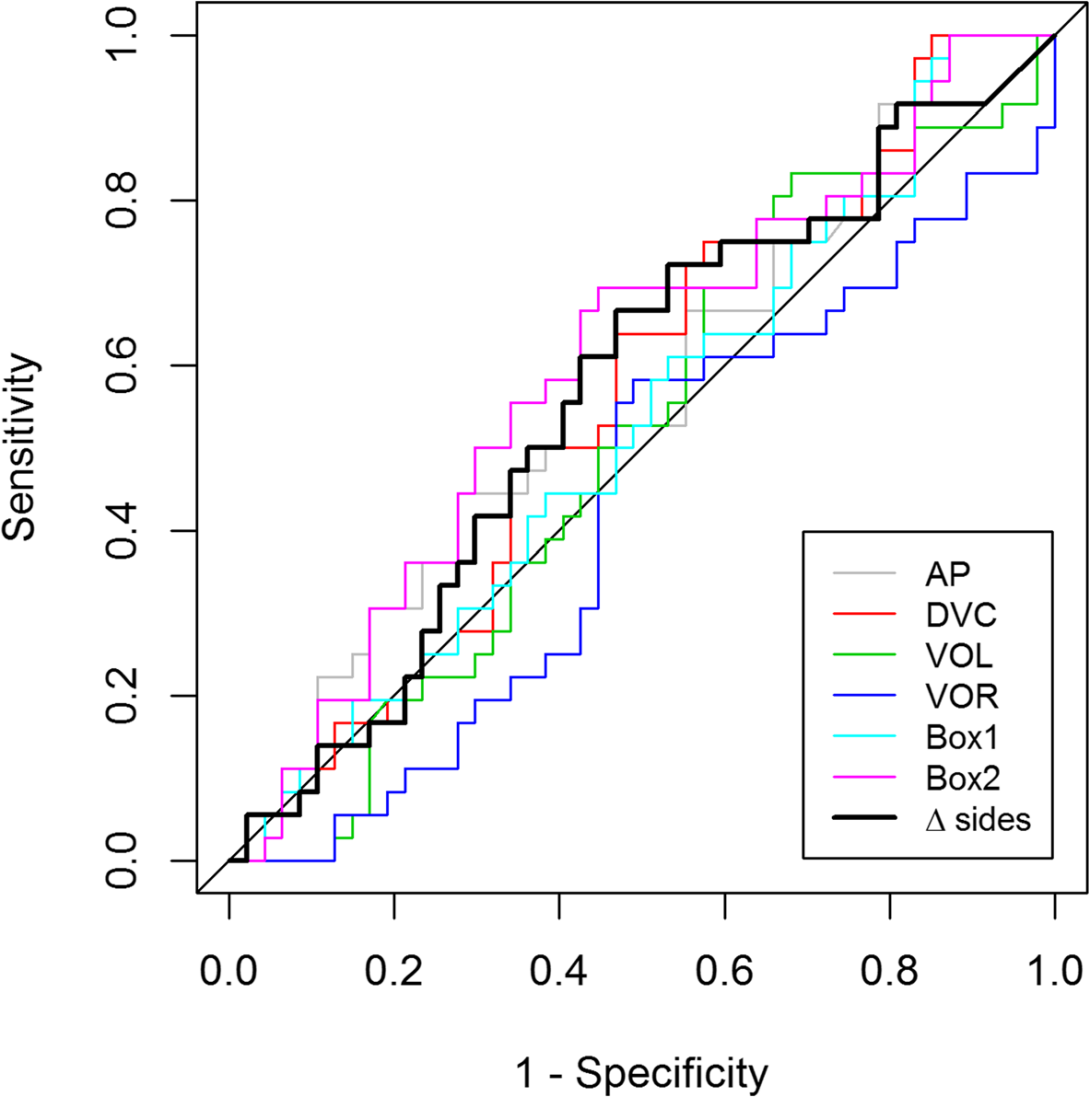
ROC Curves for Dmean doses to the Area Postrema (AP), Dorsovagal Complex (DVC), Vestibular Organ left and right (VOL/VOR), and two volumes encompassing the brainstem (Box 1 and Box 2). Also shown the curve for the difference between the left and right vestibular organ. On the x-axis specificity and on the y-axis sensitivity

**Fig. 3:**
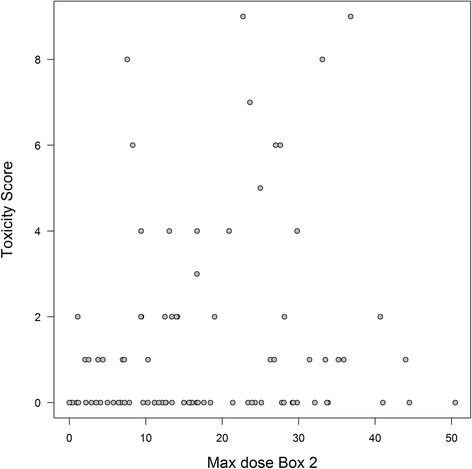
Max dose Box 2. On the x-axis radiation dose in *Gray* and on the y-axis toxicity score. Each circle is one individual measurement

In an ROC analysis no significant associations between dose and presence of toxicity (determined as a reported toxicity score larger than 0) were observed (Fig. 2, Table 2). The strongest association was observed for Box 2 (Fig. 3) with an AUC of 0.605 (95% confidence interval: 0.470 to 0.740) (Additional file 1).

**Table 2:** Area under the curve (AUC) values for delineated structures

Variables	Range	Standard error	Asymptotic 95%- confidence interval	
			Lower limit	Upper limit
D_mean_ AP	0.539	0.086	0.372	0.707
D_max_ AP	0.548	0.079	0.393	0.702
D_mean_ DVC	0.526	0.077	0.376	0.676
D_max_ DVC	0.568	0.072	0.427	0.709
D_mean_ VOL	0.424	0.080	0.266	0.581
D_max_ VOL	0.386	0.083	0.349	0.675
D_mean_ VOR	0.512	0.083	0.349	0.675
D_max_ VOR	0.503	0.082	0.342	0.664
D_mean_ Box 1	0.494	0.080	0.337	0.650
D_max_ Box 1	0.511	0.082	0.350	0.671
D_mean_ Box 2	0.569	0.078	0.416	0.721
D_max_ Box 2	0.605	0.069	0.470	0.740

Furthermore, given the hypothesis that uneven exposure to the left vestibular organ (VOL) and right vestibular organ (VOR) could cause NV toxicity, the AUC values were calculated for the difference between these two. The Dmean/Dmax values were not significantly associated with presence of NV toxicity with observed AUCs of 0.528/0.546 (95% confidence intervals: 0.350 to 0.707/0.365 to 0.728).

**Fig. 4:**
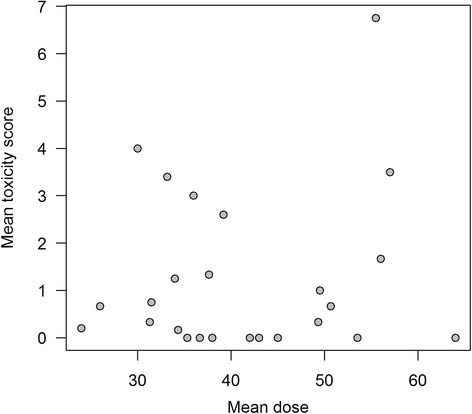
‘Between subjects’ correlation. On the x-axis is the mean radiation Dose in *Gray* and on the y-axis the mean toxicity score

**Fig. 5:**
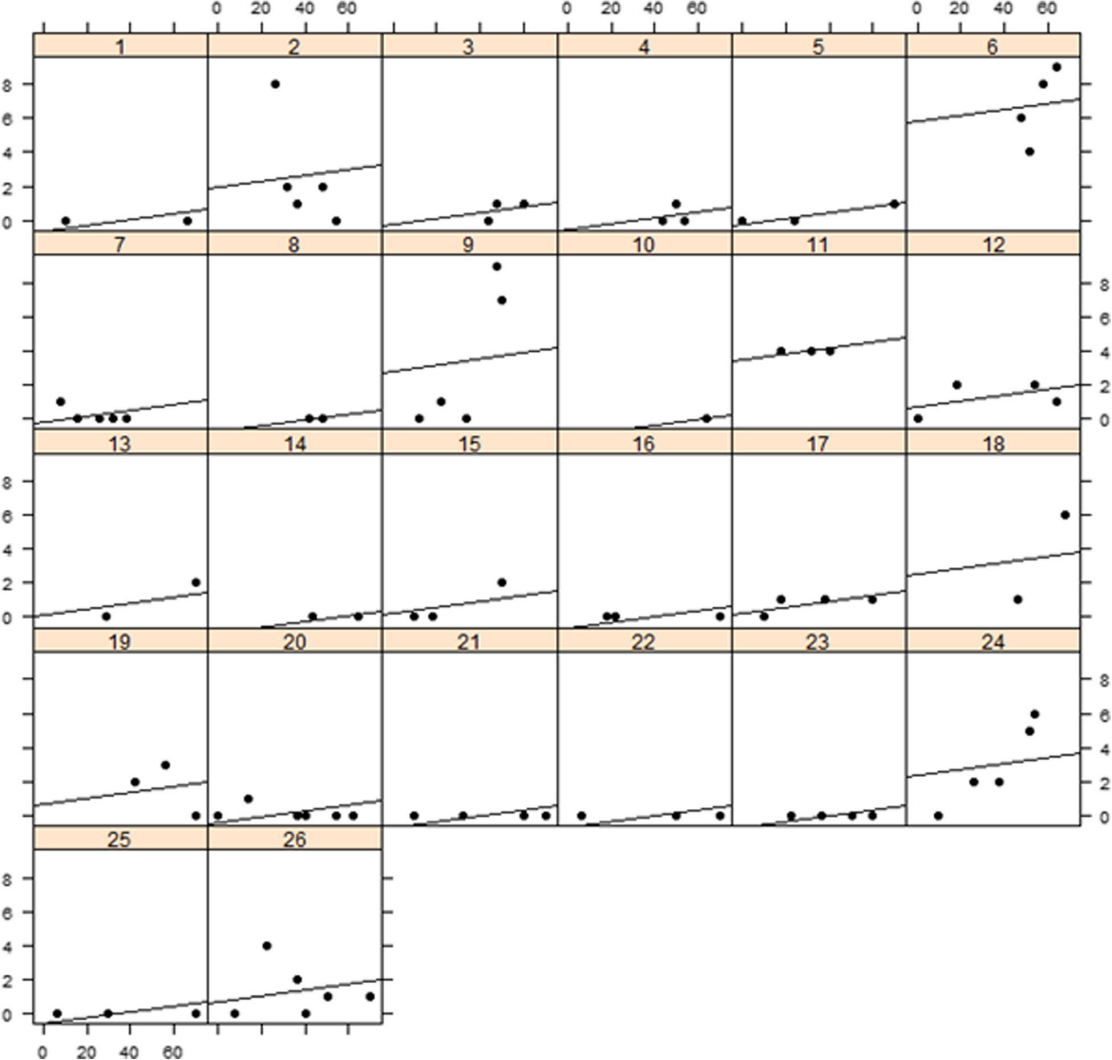
‘Within subjects’ analysis. One field/box for each individual patient (patient number above each box). On the x-axis is the radiation Dose in *Gray* and on the y-axis the toxicity score. Each dot represents one measurement of toxicity correlated to the received radiation dose at that point in time

Additionally, we observed a weak within-subjects correlation (*r* = 0.215, *p* = 0.086) and no relevant between-subjects correlation was observed (*r* = −0.069, *p* = 0.736) between administered dose and the toxicity score (Fig. 4). In a graphical illustration of repeated assessments within the same subjects at different timepoints, a strong correlation between delivered doses and toxicity scores was observed for some patients (patients 6, 9, 18 and 24), while no such association was seen in others (Fig. 5). When we looked at those patients with a higher correlation individually, we were not able to derive an overall patient characteristic where correlation showed to be stronger. Nevertheless, it is worth mentioning that none of these patients received a simultaneous integrated boost and 3 out of 4 were treated in the oropharyngeal region. A different agent of chemotherapy was given in 2 cases (Cisplatin, Paclitaxel) and the other two cases were not treated systemically.

## Discussion

IMRT is an attractive treatment option for HN cancer patients, since radiation plans have a higher conformity compared to 3D plans with improved sparing of known OAR (e.g. salivary glands) [3, 4]. Caused by inverse planning algorithms and VMAT treatment delivery some areas that were previously not exposed in former 3D plans now receive small doses of radiation which may result in additional, unknown toxicities. While some authors found a significant relation between doses to specific regions and NV toxicity, others did not.

In 2008, Monroe et al. described that dose to the DVC was linked to radiation induced nausea and vomiting (RINV). On univariate analysis they found doses to the DVC, age and T-stage as significant predictors of nausea and suggested a restricted median dose of approximately 26.9 Gy to the DVC in order to decrease incidence of RINV. The patients that had no event of nausea had a median dose of 6.56 Gy [16, 17].

Research at the MD Anderson Center in 2008 by Rosenthal et al. described a D_mean_ of the brainstem >36 Gy to be associated with RINV [9]. In the following year Ciura et al. found 82% of patients to have at least one episode of NV and stated a trend towards dose–response relationships without statistical significance [18]. Later the Monroe findings were enforced by Kocak-Uzel and colleagues, where the relation between doses to the DVC and NV reached significance at least in univariate analysis [19]. In all studies the oropharyngeal region was the predominant tumor site.

Lee et al. could not show a correlation for the AP or DVC but for the VS suggesting to limit the dose to the vestibular organs to V_40_ < 80% [11]. The relatively high doses to the VS in their study result from their patient collective exclusively containing nasopharyngeal cancers and these patients are underrepresented in most other studies, suggesting that the closer the OAR is to the tumor region and therefore to the area of higher doses the better a correlation can be shown in these small patient cohorts.

As a late complication in irradiated nasopharyngeal carcinoma survivors, vertigo has been found to be a sequel of chronic radiation otitis media and might subsequently also cause late NV [8]. Statistically, there was no significant association between chemo therapy and the presence of at least one episode of NV in our patient cohort. Exactly half of our patients received concurrent chemotherapy. Out of those, eight patients suffered from at least one episode of NV (61.5%). This is a slightly lower number than in the group of patients without concurrent systemic therapy. This might be either because in most protocols antiemetic medication was given simultaneously or simply because CTx did not cause recognizable, additional NV. Opposed to our findings in other investigations NV rates were significantly higher for patients who received concurrent CTx [9, 16, 18].

In the 4 cases with a strong correlation in our study group a different agent of chemotherapy was given in 2 cases and the other two patients were not treated systemically, indicating that chemotherapy as a likely confounder was not strong enough to cause this problem in all patients that were treated systemically or erase the correlation previously described.

In regards to age, Monroe et al. observed that younger patients seem to develop RINV more frequently than older ones [16]. This is in line with our study findings. Their reasoning was that younger patients may have an enhanced physiologic response to a variety of emetic stimulants.

Two other reports showed among age the female gender, low alcohol intake, anxiety and episodes of NV in the past as additional risk factors for developing RINV [6, 20].

Furthermore, a relation is described for the irradiated volume, the site of disease and the fractionation scheme [21].

To avoid unwanted toxicities, it has been demonstrated by Fontenla et al. that it is feasible to constrain doses to the AP and DVC without compromising target coverage. In their study they aimed to limit D_max_ to the AP and DVC to 36 and 38 Gy respectively [22]. IMRT in HN cancer patients is complex and it has been demonstrated to require a certain experience by the therapists [23–25]. Delineating structures in order to spare these and reduce toxicity is standard of practice e.g. parotid glands to avoid xerostomia [26]. Similarly, it seems reasonable to constrain dose to the dorsal vagal complex for radiotherapy planning [27].

Clearly there are limitations to our study; all pharyngeal tumor sites were included in our study resulting in a variety of dose deposition on OARs and apparently consecutively a lower/ higher likelihood of adverse effects. Other possible confounder were the administration of chemotherapy and prophylactic antiemetic medication. Furthermore, statistic evaluation proved to be challenging, as there were numerous points in time where no toxicity was reported and due to several measurements per patient, data were observed at different timepoints were not independent and standard statistic workups were not applicable.

A previous investigation of Rathod et al. indicated that IMRT results in clinically meaningful and statistically better QoL scores compared to 3D techniques [28]. Evaluation of patient’s quality of life (QoL) as well as psychological factors, that could lead to NV, should potentially also be included in future studies.

## Conclusions

RINV for HN patients is one of the most frequent acute toxicities with more than half of the patients being affected during treatment and the knowledge of the underlying mechanisms remains sparse.

No significant within subjects correlation between doses delivered to the areas in question and higher toxicity scores was observed (*r* = 0.215, *p* = 0.086).

NV was more frequent when treating the oropharyngeal region, as well as if patients were female and/or of a younger age.

RINV as a multifactorial process is influenced by patient’s characteristics as well as tumor site and treatment planning. As IMRT is able to spare organs at risk if they are previously defined and delineated, our study seems to support previous investigations that recommended dose avoidance for subareas of the brainstem in order to avert unnecessary side effects for patients. Small safety margins and daily image guidance will also help to avoid unnecessary doses to OARs and spare patients from side effects.

Nevertheless, no conclusive or resounding correlation could be found in our investigation suggesting strongly not to compromise on target coverage when treating HN cancer patients.

## Additional file

Additional file 1: ROC curves with cis. (TIF 75 kb)

## Abbreviations

AP: Area postrema
AUC: Areas under the receiver operating characteristics curve
BS: Brainstem
CTCAE: Common toxicity criteria
CTx: Chemotherapy
DVC: Dorsovagal complex
HN: Head and neck
IMRT: Intensity modulated radiation therapy
NV: Nausea and vomiting
OAR: Organs at risk
PTV: Planning target volume
RINV: Radiation induced nausea and vomiting
ROC: Receiver operating characteristics
VMAT: Volumetric modulated arc treatment
VOL: Left vestibular organ
VOR: Right vestibular organ
VS: Vestibular system
