# Supplementary figures and images for: Correlation between delivered radiation doses to the brainstem or vestibular organ and nausea & vomiting toxicity in patients with head and neck cancers – an observational clinical trial
Source: Radiat Oncol. 2017 Jul 4;12:113. doi: 10.1186/s13014-017-0846-4 (PMC5496249; doi:10.1186/s13014-017-0846-4)

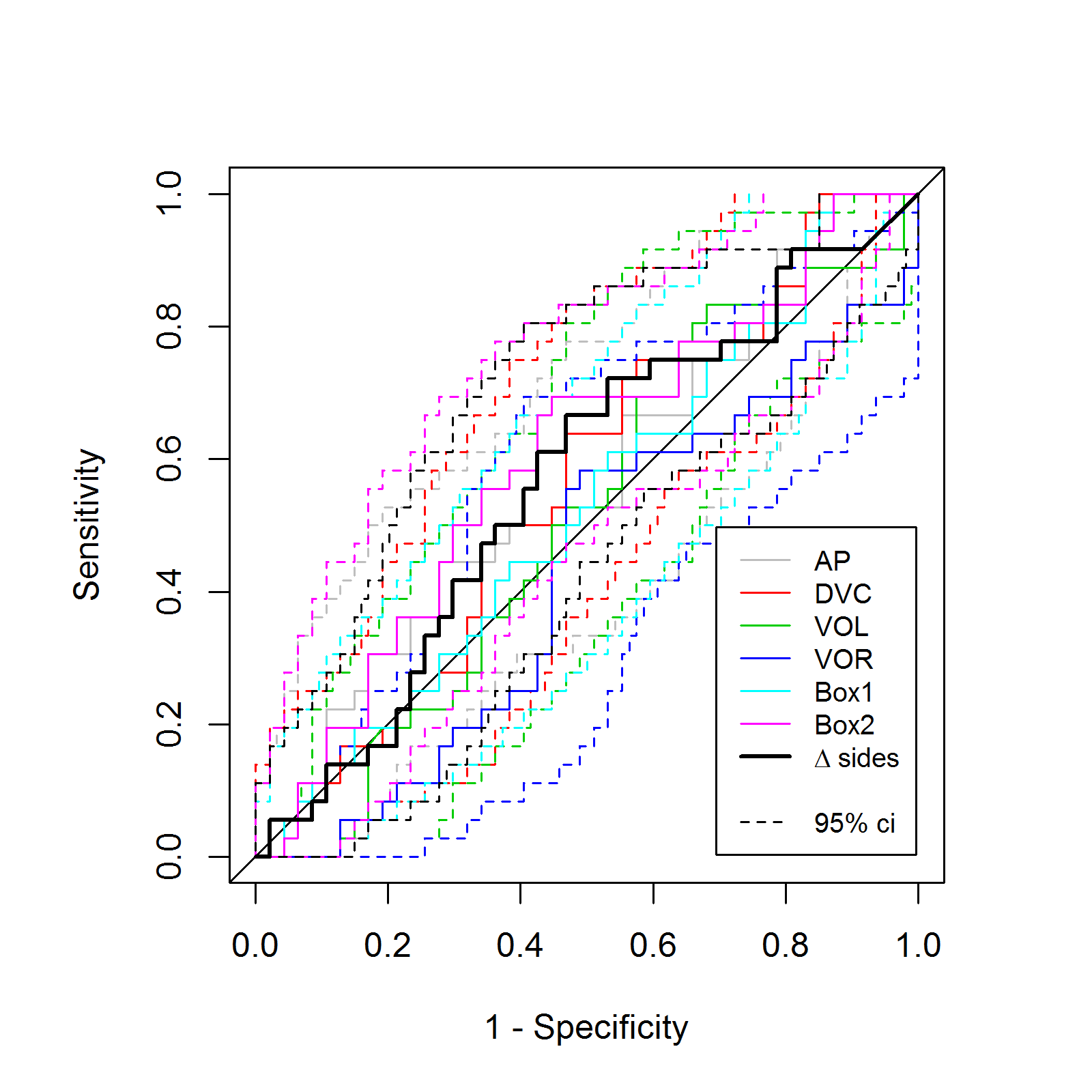

Supplement: Additional file 1: — ROC curves with cis. (TIF 75 kb) [file 13014_2017_846_MOESM1_ESM.tif]
